# Cadherin-11 in Renal Cell Carcinoma Bone Metastasis

**DOI:** 10.1371/journal.pone.0089880

**Published:** 2014-02-24

**Authors:** Robert L. Satcher, Tianhong Pan, Chien-Jui Cheng, Yu-Chen Lee, Song-Chang Lin, Guoyu Yu, Xiaoxia Li, Anh G. Hoang, Pheroze Tamboli, Eric Jonasch, Gary E. Gallick, Sue-Hwa Lin

**Affiliations:** 1 Orthopedic Oncology, University of Texas, MD Anderson Cancer Center, Houston, Texas, United States of America; 2 Translational Molecular Pathology, University of Texas, MD Anderson Cancer Center, Houston, Texas, United States of America; 3 Genitourinary Medical Oncology, University of Texas, MD Anderson Cancer Center, Houston, Texas, United States of America; 4 Pathology, University of Texas, MD Anderson Cancer Center, Houston, Texas, United States of America; 5 Department of Pathology, School of Medicine, College of Medicine, Taipei Medical University, Taipei, Taiwan; 6 Department of Pathology, Taipei Medical University Hospital, Taipei, Taiwan; Rutgers - New Jersey Medical School, United States of America

## Abstract

Bone is one of the common sites of metastases from renal cell carcinoma (RCC), however the mechanism by which RCC preferentially metastasize to bone is poorly understood. Homing/retention of RCC cells to bone and subsequent proliferation are necessary steps for RCC cells to colonize bone. To explore possible mechanisms by which these processes occur, we used an *in vivo* metastasis model in which 786-O RCC cells were injected into SCID mice intracardially, and organotropic cell lines from bone, liver, and lymph node were selected. The expression of molecules affecting cell adhesion, angiogenesis, and osteolysis were then examined in these selected cells. Cadherin-11, a mesenchymal cadherin mainly expressed in osteoblasts, was significantly increased on the cell surface in bone metastasis-derived 786-O cells (Bo-786-O) compared to parental, liver, or lymph node-derived cells. In contrast, the homing receptor CXCR4 was equivalently expressed in cells derived from all organs. No significant difference was observed in the expression of angiogenic factors, including HIF-1α, VEGF, angiopoeitin-1, Tie2, c-MET, and osteolytic factors, including PTHrP, IL-6 and RANKL. While the parental and Bo-786-O cells have similar proliferation rates, Bo-786-O cells showed an increase in migration compared to the parental 786-O cells. Knockdown of Cadherin-11 using shRNA reduced the rate of migration in Bo-786-O cells, suggesting that Cadherin-11 contributes to the increased migration observed in bone-derived cells. Immunohistochemical analysis of cadherin-11 expression in a human renal carcinoma tissue array showed that the number of human specimens with positive cadherin-11 activity was significantly higher in tumors that metastasized to bone than that in primary tumors. Together, these results suggest that Cadherin-11 may play a role in RCC bone metastasis.

## Introduction

Renal cell carcinoma (RCC) often metastasizes to bone, lymph nodes, liver, lung, and brain [1]. Bone metastases are painful are associated with a high incidence of pathologic fractures due to their almost exclusive osteolytic behavior [2], [3]. RCC bone metastases are also relatively resistant to radio- and chemo- therapy [4], [5]. Although the management of bone metastases has been significantly improved by the addition of anti-angiogenic agents, most patients eventually develop resistance to these therapies. Surgical resection of RCC bone metastasis remains challenging due to induced vascularity, and a propensity to recur if complete resection is not possible [6], [7]. Consequently, the prognosis for RCC patients who develop bone metastases is dismal, with a mean survival of 12 months [3], [5]. A better understanding of the factors that play a role in RCC bone metastasis could result in preventive/therapeutic strategies that might be effective in prolonging patients’ survival.

The molecular mechanisms by which RCC metastasizes to the bone are not fully understood. Tumors are heterogeneous and include cells with the ability to metastasize preferentially to numerous organ sites [8]. Once cancer cells dislodge from the primary site and survive in the circulation, they must intravasate and grow at a metastatic site [9]. For RCC cells to develop metastatic colonies in the bone, a series of critical processes must occur, including survival in circulation, homing, retention, and proliferation in the bone microenvironment.

Many alterations in tumor cells may be required for successful bone metastases, including altered expression of adhesion factors. The adhesion molecule Caderin-11 (Cad11), a calcium-dependent cell-cell adhesion molecule and mesenchymal marker, was originally identified from mouse osteoblasts [10], and is the most abundant cadherin present in human osteoblasts [11]. Recent studies have demonstrated numerous critical roles for Cad11 in the formation of bone metastasis in prostate cancer [12], [13], [14] and breast cancer [15]. In addition, CXCR4, the receptor for chemokine stromal cell derived factor 1α (SDF-1α), has been reported to mediate homing to bone in prostate and breast cancer cells [16], [17]. Whether these membrane proteins are involved in RCC bone metastasis has not been studied.

Following metastatic cell homing/retention in bone, the progression of RCC in bone is likely mediated by a series of interactions between invading tumor cells and the bone microenvironment [18], [19]. Angiogenesis is required, and studies have confirmed that hypervascularity is commonly associated with RCC [6], [7]. The loss of the von Hippel-Lindau (VHL) tumor suppressor gene in most of RCCs leads to constitutive activation of hypoxia-inducible factor-1α (HIF-1α), resulting in the induction of multiple pro-angiogenic molecules such as vascular endothelial growth factor (VEGF) [7], [20], [21]. Moreover, tumor-induced osteolysis and the subsequent release of factors from bone, further enhance tumor growth by creating a vicious cycle that promotes tumor growth in the bone [22], [23].

In this study, we generated bone-tropic and non-bone tropic 786-O RCC cell lines from human 786-O cells via intracardiac injection of SCID mice and identified molecules that may be involved in the metastasis of RCC to bone. Our analyses suggest that Cad11 is an important mediator of 786-O bone metastasis formation. Specifically, we found that Cad11 expression is increased in 786-O cells derived from bone as compared to parental, liver, or lymph node-derived cells. Evidence for the functional impact of this increased expression is also demonstrated.

## Materials and Methods

### Ethics Statement

All experimental procedures involving animals were approved by UT M D Anderson’s Animal Care and Use Committee. All the experiments involving human tissue samples were approved by the UT MD Anderson Cancer Center Clinical Research Committee and the UT MD Anderson Cancer Center Institutional Review Board (IRB). All participants signed written consent to permit tissue use in research studies as part of their clinical trials consent process. Patient consent is recorded in a central database managed by the Office of Protocol Research at UT MD Anderson Cancer Center. This consent procedure is approved by the UT MD Anderson Cancer Center Office of Protocol Research.

### Animals

Severe combined immunodeficient (SCID) mice (male, 5-week-old) were purchased from Jackson Laboratory and maintained in M. D. Anderson’s animal facilities. All experimental procedures involving animals were performed in compliance with institutional requirements and approved by M. D. Anderson’s Animal Care and Use Committee. A total of 80 SCID mice were approved for this study.

### Parental and Organ-derived Cell Lines

The human 786-O RCC cell line, derived from a primary clear cell renal adenocarcinoma, was purchased from the American Type Culture Collection (ATCC; Manassas, CA). To express luciferase in 786-O RCC cells for *in vivo* imaging, human 786-O RCC cells were transduced with a bi-cistronic retroviral vector containing cDNA encoding for luciferase (Luc) and green fluorescent protein (GFP) genes (pBMN-Luc-I-GFP plasmid) as described previously [12]. Transduced cells were further sorted by fluorescence-activated cell sorting (FACS) based on GFP positivity and termed parental 786-O RCC cells.

To establish organ-derived cell lines, we injected parental 786-O cells intracardially into SCID mice. Briefly, parental 786-O cells were harvested from subconfluent cell culture flasks. A total of 50 µl cell suspension containing 1×10^6^ cells in PBS was injected into the left ventricle of SCID mouse. The dissemination of tumor cells in mouse was determined by bioluminescence imaging (BLI) with an IVIS 200 Imaging System (Xenogen). Nine weeks after injection, mice were killed, and tumor cells in various organs were isolated. For tumors in the hind limbs, the femurs were flushed with 10 ml of RPMI culture medium containing 10% heat-inactivated fetal bovine serum (FBS). The cells flushed from the bone marrow and the rest of the bone, which were chopped into pieces, were cultured in vitro. For tumors grown in liver and lymph nodes, the affected tissues were taken out, cut into pieces, and cultured in the medium as described above. After culturing for several weeks, populations of bone-derived 786-O (Bo-786-O), liver-derived 786-O (Liv-786-O) and lymph node-derived 786-O (LN-786-O) RCC cells were obtained. All the parental and organ-derived 786-O RCC cells were cultured at 37°C with 5% CO_2_ in RPMI medium (Invitrogen) containing 10% FBS.

### Quantitative RT-PCR

Total RNA was extracted from cells using RNeasy mini purification kit (Qiagen, Valencia, CA) according to the manufacturer’s protocol. Single-strand cDNA was synthesized from 1.0 µg of total RNA using TaqMan Reverse Transcription Reagents (Life Technologies). Real-time PCR was performed in Multiplex Quantitative PCR System (STRATAGENE, Model Mx3000pTM) with each reaction containing 0.4 µM primers, 1× Sybr Green PCR Super Mix (Applied Biosystem) and 20 ng of cDNA template. The thermal cycling condition for PCR was 95°C for 10 min followed by 40 cycles at 95°C for 15 sec, 60°C for 1 min per cycle. The value of threshold cycle (Ct) was generated at every cycle during a run. Messenger RNA levels were compared to *β*-actin for standardization of samples. The expression of gene-of-interest was determined by the formation of 2^-delta Ct^ as reported previously [12], [24].

Primers used for real time PCR analysis were selected according to previous publications or by using primer 3 and BLAST system (NCBI). The nucleotide sequences of the primers are shown in Table S1.

### Western Blot Analysis

Total protein was extracted from cells using mammalian tissue lysis/extraction reagent (Sigma, St. Louis, MO) supplemented with protease inhibitor cocktails according to the manufacturer’s protocol. Equal amounts of protein (50 µg/lane) were loaded and separated on 4–12% SDS−polyacrylamide gel electrophoresis (PAGE) gel. Protein was transferred onto a nitrocellulose membrane and probed with anti-Cad11 (Invitrogen), anti-CXCR4 (1∶1000; EMD Millipore, Billerica, MA), or anti-*β*-actin (1∶2000; Santa Cruz Biotechnology) antibody. Membranes were then incubated with horseradish peroxidase-conjugated anti-mouse, anti-rabbit or anti-goat IgG, and the proteins were visualized with ECL detection kit (Pierce Biotechnology). Image J software was used for densitometry analysis to quantify protein levels.

### Flow Cytometry

Cells were harvested by trypsin digestion and 2×10^6^ cells in PBS was incubated with anti-Cad11 antibody mAb2C7 [25] or human CXCR4 antibody (2.5 µg/10^6^ cells; R&D System) on ice for 45 min. After washing two times, cells were incubated with Alexa fluor 647 (AF647)-conjugated donkey anti-mouse IgG (1∶1000; Jackson ImmunoResearch, Laboratories, INC) on ice for another 45 min in the dark. Stained cells were then washed and suspended in 350 µl 1%BSA/PBS buffer and fluorescence activated flow cytometry analyses were performed on a FACScan flow cytometer (Beckman Coulter “Gallios”).

### Immunofluorescence

Cells (3×10^4^) were seeded into 24-well plate with cover slip for 48h followed by fixation with cold methanol for 10 min. After washing, cells were incubated with blocking solution containing 2% normal donkey serum, 1% bovine serum albumin, and 0.01% Triton X-100 in PBS for 30 min at room temperature (RT) followed by the incubation with mouse anti-Cad11 antibody (Invitrogen) overnight at 4°C. Mouse IgG (Santa Cruz Biotechnology) was used as a negative control. On the second day, after washing, cells were incubated with Alexa Fluo (AF) 594-conjugated donkey anti-mouse secondary antibody (1∶500, Jackson ImmunoResearch) for 45 min at RT followed by staining with 4′,6 diamidino-2-phenylindole (DAPI) for 10 min. Cells were then mounted with Vectashield mounting medium and sealed with a nail gel. Images were acquired using an OLYMPUS confocal microscope (FV10-ASW3.0 Viewer).

### Cell Proliferation Assay

Cells were seeded in 6-well plate with each containing 8×10^4^ cells in 3 ml of culture medium. The number of cells was counted daily for 4 days with a hemocytometer.

### Cell Migration Assay

Cells (2×10^4^) in 300 µl of serum-free RPMI1640 were seeded into FluoroBlock TM Cell Culture insert (BD Falcon). The lower chamber of a 24 well plate (BD Falcon) contained 500 µl of pre-warmed 0.5% FBS RPMI culture media. Five hours after seeding, the non-migrating cells remaining in the insert were scraped off using cotton scrub and the migrated cells in the bottom part of the insert were labeled with calcein AM in 0.5% FBS RPMI medium. Cells that migrated through the membranes were quantified by determining cell number in five randomly chosen visual fields at ×100 magnification.

### Knockdown of Cadherin-11

To knock down Cad11 in Bo-786-O cells, the lentiviral vector containing cadherin-11 shRNA plasmid (pLKO.1-puro, Sigma-Aldrich, TRCN0000303363) was co-transfected with the packaging plasmid pCMV-dR8.2 dvpr and the envelope plasmid pCMV-VSVG into 293FT cells using Lipofectamine 2000 (Invitrogen). The lentiviral vector containing non-targeting shRNA (pLKO.1 puro, Sigma-Aldrich) was used as a negative control. The culture medium containing the lentivirus was collected in 48 h, filtered and used to infect Bo-786-O cells in the presence of 8 µg/ml polybrene (Sigma). Twenty-four hours after infection, medium was replaced with fresh medium containing 0.25 µg/ml puromycin for selecting stable Cad11 knockdown cells (Bo/shCad11) or stable non-targeting shRNA control cells (Bo/shCont).

### Immunohistochemical Staining of Human RCC Specimens

A tissue microarray (TMA) containing RCC specimens from primary tumors and bone metastasis was immunostained with anti-Cad11 antibody (R&D System) using the procedures described previously [12]. The reactivity of Cad11 in the tumor cells was marked as “P”, “W”, “N” for strong positivity (in either membrane and cytoplasm staining), weak positivity, and negative, respectively. There were three cores per sample. If one or more cores were positive, the case was graded as positive. Otherwise the case was graded as negative. A total of 41 samples from primary RCC tumor and 26 samples from RCC bone metastasis were evaluated for Cad11 expression. All the experiments involving human tissue samples were performed in compliance with Institutional requirements and approved by Institutional Review Board (IRB).

### Statistical Analysis

All data were collected from three or more independent experiments and values were expressed as mean ± SE. Statistical significance was assessed by students *t* test or by chi-square analysis. The level of significance was set at *p*<0.05.

## Results

### Establishment of Organ-derived 786-O Cell Lines

**Figure 1:**
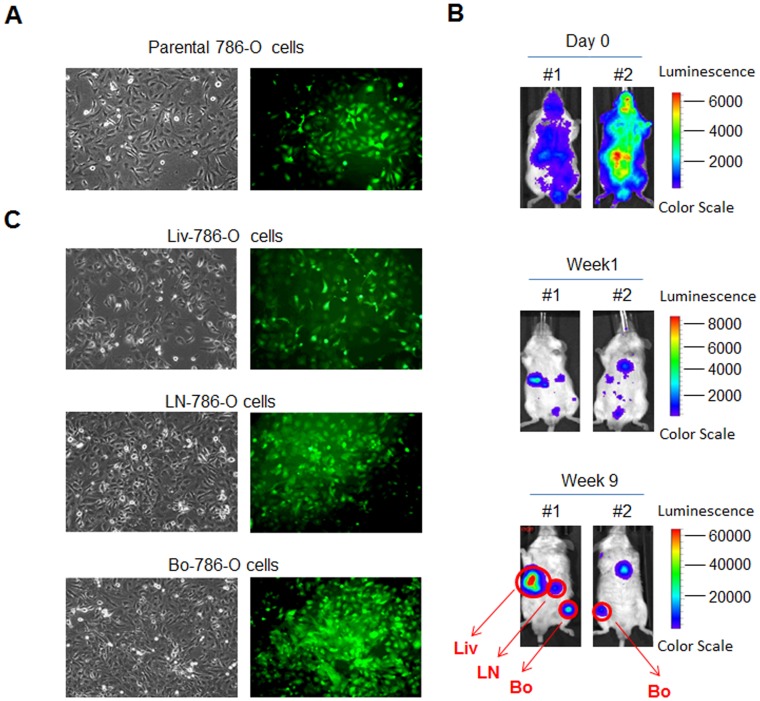
Generation of organ-tropic 786-O RCC cells. (A) Parental 786-O RCC cells were labeled with luciferase and GFP. (B) Images of bioluminescence of mice at indicated time point after intracardiac injection with parental 786-O cells. (C) 786-O cells derived from liver, lymph node and bone, were GFP-positive.

Luciferase-labeled 786-O RCC cells that were also GFP-positive in in vitro cultures (Fig. 1A) were injected intracardially into mice. After 5 minutes, a marked whole body bioluminescence signal was observed (Fig. 1B, day 0), indicating that the injected parental cells were disseminated throughout the mice. After one week, the bioluminescence signals subsided and appeared at specific sites (Fig. 1B). After nine weeks, strong bioluminescence signals were observed in the hind legs as well as a few other organs (Fig. 1B), indicating that a fraction of parental 786-O cells disseminated to multiple tissues and grew at these sites. Tumor cells were then isolated from affected organ sites, including liver, lymph nodes, and femur, and cultured in vitro to generate liver-derived (Liv-), lymph node-derived (LN-) and bone-derived (Bo-) 786-O RCC cells, respectively. All organ-derived 786-O cells were positive for GFP (Fig. 1C), indicating that these cells were from parental 786-O tumor cells.

### Expression of Cad11 in Organ-derived 786-O Cell Lines

**Figure 2:**
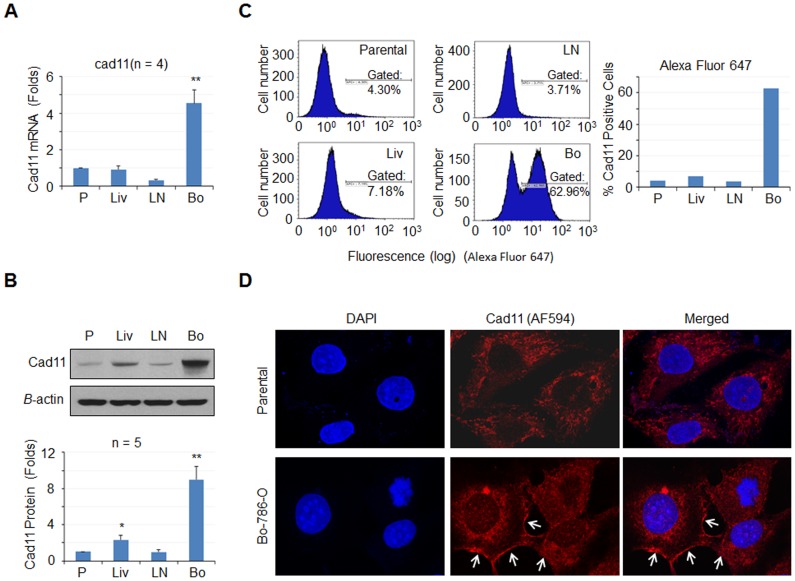
Expression of Cad11 in 786-O cell lines derived from metastases to various organs. (A) Quantitative PCR for the message levels of *Cad11* in the four 786-O cell lines. (B) Western blotting for the protein levels of Cad11 in four 786-O cell lines. Upper panel: A representative image of Western blot. Lower panel: Quantification of band density using Image J software. Data were expressed as folds of parental 786-O cells and the values were the Mean ± S.E. n = 5. *: *p*<0.05; **: *p*<0.01 as compared to parental 786-O cells. (C) FACS for surface expression of Cad11 in the four 786-O cell lines. Data were expressed as percentage of gated cells. (D) Immunofluorescence staining of cells with anti-Cad11 antibody. P, Parental 786-O; Liv, Liv-786-O; LN, LN-786-O, and Bo, Bo-786-O RCC cells.

Because the Cadherin11 (Cad11) adhesion molecule has been reported to be involved in bone metastasis of prostate [12] and breast [15] cancers, we examined its expression in parental 786-O cells and three organ-derived cells by real-time PCR. *Cad11* gene expression was increased 4.6±0.6 fold in Bo-786-O cells compared to that in parental 786-O cells (Fig. 2A). In contrast, *Cad11* message was not increased in Liv-786-O or LN-786-O cells compared to the parental cells (Fig. 2A). Western blotting for Cad 11 revealed a single band of apparent molecular weight ∼100 kDa in all four cell lines (Fig. 2B, upper panel). Densitometry analysis showed that the protein levels of Cad11 were significantly increased (9.0±1.3 fold) in Bo-786-O cells relative to expression in parental cells (Fig. 2B). Expression of Cad11 protein was also increased in Liv-786-O cells (2.3±0.4 fold) compared to that in parental cells (Fig. 2B). To examine whether the Cad11 was targeted to plasma membrane, we conducted FACS analysis using anti-Cad11 antibody mAb 2C7, which recognizes the extracellular domain [25]. We found that 63% of Bo-786-O cells were positive with Cad11, while only 4.3%, 7.2%, and 3.7% were positive with Cad11 in parental 786-O, Liv-786-O, and LN-786-O cells, respectively (Fig. 2C). Interestingly, FACS analysis revealed two populations of cells in Bo-786-O cells: one population (63%) of cells was Cad11-positive, whereas another population (37%) of cells was Cad11-negative, suggesting that Cad11 expression is increased in a subset of 786-O cells that metastasized to bone. Immunofluorescence staining of parental and Bo-786-O cells showed that more Cad11 protein was localized on plasma membrane of Bo-786-O cells when compared to that in parental 786-O cells (Fig. 2D). Together, these observations suggest that Cad11 expression is higher in 786-O cells that metastasized to bone relative to 786-O cells that metastasize to other organ sites.

### Expression of CXCR4 in Organ-derived 786-O Cell Lines

**Figure 3:**
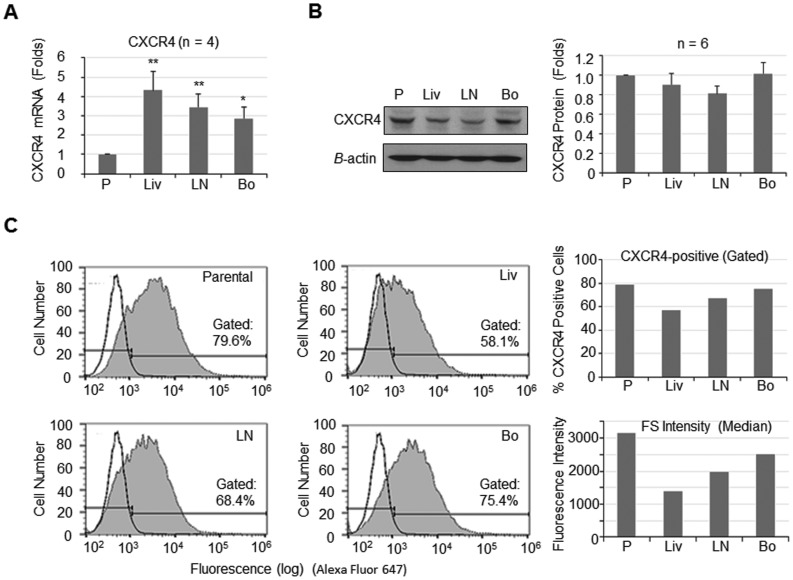
Expression of CXCR4 in 786-O cell lines. (A) Quantitative PCR for the message levels of *CXCR4* in the four 786-O cell lines. *: *p*<0.05; **: *p*<0.01 as compared to parental 786-O cells. (B) Western blotting for the protein levels of CXCR4. Left panel: A representative image of Western blot. Right panel: The quantification of band density. Data were expressed as folds ± S.E. of parental 786-O cells. n = 6. (C) The number of cells with CXCR4 expression in cell surface was determined by FACS. Data were expressed as percentage of gated cells (right upper panel) and as the median of fluorescence intensity (right lower panel).

Previous studies have shown that the chemokine receptor CXCR4 plays a role in breast and prostate cancer bone metastases via interactions with its ligand SDF-1 [16], [17]. We therefore examined the levels of *CXCR4* in the four 786-O cell lines. Quantitative PCR analysis showed that the message levels of *CXCR4* was significantly increased in the three organ-derived 786-O cells compared to parental 786-O cells, with 4.3±0.9 (p<0.01), 3.4±0.6 (p<0.01) and 2.8±0.5 (p<0.05) fold increases in Liv-786-O, LN-786-O and Bo-786-O cells, respectively (Fig. 3A). However, no significant differences in the levels of CXCR4 protein were observed among these cell lines (Fig. 3B). Consistent with the results from Western blot, FACS analysis showed that the number of CXCR4-positive cells and the fluorescence intensity were high in all the four cell lines (Fig. 3C). However, no significant difference was observed amongst them (Fig. 3C). The reason for the inconsistency between the CXCR4 message and protein levels in the 786-O cell lines is not clear. These observations indicated that CXCR4 may play a critical role in metastasis, but not specifically to the bone.

### Expression of Angiogenic and Osteolytic Factors in Organ-derived 786-O Cell Lines

**Figure 4:**
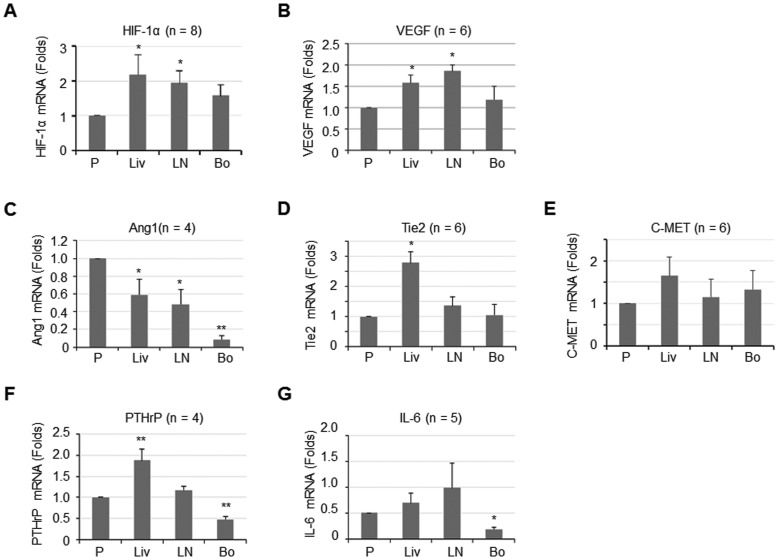
Message levels of angiogenic and osteolytic factors in 786-O RCC cell lines. Quantitative PCR for the message levels of angiogenic factors *HIF-1α* (A), *VEGF* (B), *Ang1* (C), *Tie-2* (D) and *c-Met* (E), and osteolytic factors *PTHrP* (F) and *IL-6* (G) in the four 786-O cell lines. Data were expressed as folds of parental 786-O cells and the values were the Mean ± S.E. *: *p*<0.05; **: *p*<0.01 as compared to parental 786-O cells.

Many factors may contribute to metastatic progression of RCC in bone. RCC bone metastases are typically hypervascular [6], [7]. Thus, we examined whether the expression of angiogenic factors is increased in 786-O cells that metastasized to bone. HIF-1α, VEGF, endothelium-specific receptor tyrosine kinase Tie-2, and angiopoeitin-1 (Ang-1), a ligand for Tie-2 [26], are candidate angiogenic factors. c-MET is a transmembrane receptor tyrosine kinase that has been reported as a proto-oncogene, increased expression of which is associated with poor pathologic features and poor prognosis in RCC[27], [28]. As shown by real time PCR analysis, we found that the message levels of *HIF-1α and VEGF* were significantly higher in Liv-786-O and LN-786-O cells than that in parental cells (Fig. 4A; 4B). However, the levels of *HIF-1α and VEGF* message in Bo-786-O cells were not significantly different from those in parental 786-O cells (Fig. 4A, B). The levels of *Tie2* and *c-MET* in Bo-786-O were also similar to those in parental 786-O (Fig. 4D, E). Interestingly, we found that *Ang-1* gene expression was significantly lower in organ-derived cell lines, with the Bo-786-O cells showing the most significant decrease compared to the parental 786-O cell line (Fig. 4C).

RCC bone metastases are characteristically osteolytic [2], [3]. Tumor-induced osteoclastic activity has been shown to release factors that are critical for the metastatic growth of RCC in bone [22], [23]. PTHrP and IL-6 are both important factors for modulating bone metabolism and osteoclastic activity [29], [30], [31]. RANKL is known to play a role in osteolytic bone remodeling [29], [30]. We therefore determined the expression of *PTHrP, IL-6* and *RANKL* in these organ-derived cell lines. Real time PCR assay showed that the levels of *PTHrP* and *IL-6* message were significantly lower, about 0.5 (p<0.01) and 0.4 (p<0.05) fold, respectively, in Bo-786-O cells compared to those in parental cells (Fig. 4F, 4G). While RANKL is an important factor contributing to osteoclast activation, the levels of RANKL in 786-O cells were too low to be detected (data not shown).

### Effects of Cad11 on the Cell Proliferation and Migration

**Figure 5:**
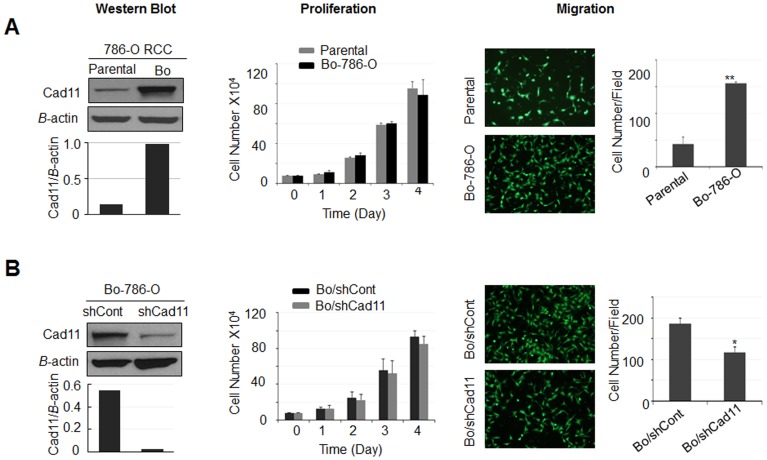
Effect of Cad11 on cell proliferation and migration of Bo-786-O cells. (**A**) Proliferation and migration of parental and bone-derived 786-O cells. Left: Western blot of Cad11 protein. Middle: Cell proliferation. Right: Cell migration. A representative image of cell migration and the quantification of cells that migrated to the other side of migration inserts were shown. Values for migration were expressed as the average of migrated cells per microscope field (X100). (B) Effect of Cad11 knockdown on the proliferation and migration of Bo/shCont and Bo/shCad11 cells.

Next, we examined proliferation and migration between parental and bone-derived 786-O cells. Consistent with the results in Fig. 2, the Cad11 protein level is about 7 fold higher in Bo-786-O than in parental 786-O cells as determined by Western blot assay (Fig. 5A). There was no significant difference in the proliferation between these two cell lines (Fig. 5A). However, the number of migrated cell was more in Bo-786-O cells than that in parental 786-O cells (p<0.01) (Fig. 5A).

We further examined whether Cad11 played a role in the increased migration of Bo-786-O cells via a knockdown model. For these experiments, we established stable Bo/shCad11 cell line, in which Cad11 was suppressed by specific Cad11-targeting shRNA (Fig. 5B). As shown by Western blot, the Cad11 protein level in Bo/shCad11 cells was decreased by 95% as compared to the control Bo/shCont cells (Fig. 5B). Reduction in Cad 11 had no significant effects on cell proliferation rate (Fig. 5B). However, the migration of Bo/shCad11 cells was significantly slower (p<0.05) than that in Bo/shCont control cells (Fig. 5B). The results that suppression of Cad11 resulted in the decrease of cell migration in Bo-786-O cells indicate that Cad11 contributes to the increased migration seen in bone-derived 786-O cells.

### Cad11 Expression in Human RCC Specimens

**Figure 6:**
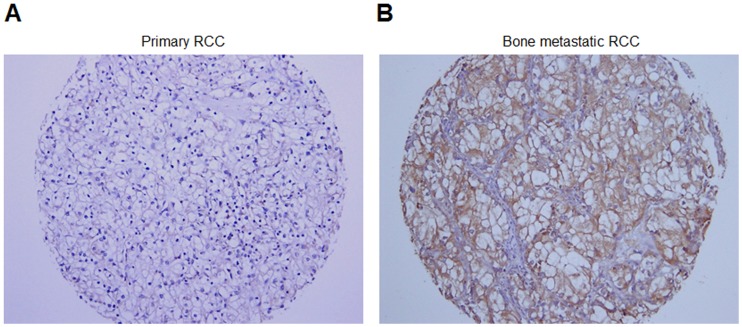
Immunohistochemical staining of human RCC specimens with anti-Cad11 antibody. (A) Representative image of primary RCC; (B) Representative image of bone metastatic RCC.

To examine whether increases in Cad11 in bone metastasis also occur in clinical specimens, we conducted immunohistochemical staining of Cad11 in a human renal carcinoma tissue array. A total of 41 specimens from primary tumors and 26 specimens from bone metastasis were evaluated for Cad11 expression. About 20% (8 of 41) of primary tumors examined were positive for Cad11, whereas 46% (12 of 26) of bone metastasis specimens were positive for Cad11 (p<0.02, chi square test) (Fig. 6, Table 1). Thus, Cad11 expression increases in RCC bone metastasis compared to that in primary tumors. Because Cad11 contributes to the migration of bone-derived 786-O cells (Fig. 5B), the increase of Cad11 expression in RCC bone metastasis suggests that Cad11 may play a role in RCC bone metastasis.

**Table 1:** Cad11 expression in human primary RCC and bone metastatic RCC specimens.

Type of Specimen	Total No. of Samples	Cadherin-11-Positive		P*
		No. of Samples	%	
Primary RCC	41	8	8/41 (20%)	
RCC Bone Metastases	26	12	12/26 (46%)	0.02

Staining of human RCC samples for cadherin-11.

*: chi-square analysis.

## Discussion

Organ-specific metastasis has been observed for many cancers; however, the mechanisms that confer organ specificity are only beginning to be understood. Our study provides an approach to address factors critical for bone-specific metastasis. We identified Cad11 as one of the molecules that is upregulated in bone-derived, but not in lymph node or liver-derived 786-O cells. In addition, we showed that knockdown of Cad11 expression in Bo-786-O cells decreased their migration, but not proliferation. Cad11 is a mesenchymal cell adhesion molecule and is the major cadherin family protein expressed in osteoblasts, although lower levels of Cad11 message can be detected also in brain, lung and heart [32]. Thus, Cad11 may contribute to bone metastasis through increasing RCC cell migration or the adhesion of RCC with the osteoblasts present in the bone marrow. As metastasis is a multi-step process, it is likely that many other factors contribute to metastatic progression of RCC in bone. Indeed, FACS analysis showed that there were two populations of cells in Bo-786-O cells: one population (63%) of cells that was Cad11-positive and another population (37%) of cells that was Cad11-negative (Fig. 2C). These observations suggest that factors other than Cad11 are also involved in the metastasis of 786-O cells to bone.

Increases in Cad11 expression in Bo-786-O cells may be due to epithelial-mesenchymal transition (EMT). This possibility is supported by recent studies indicating that cadherins play important roles in the process of EMT during both normal embryonic development and cancer progression [33], [34], [35]. During tumor progression in breast, prostate, gastric, and pancreatic cancers [36], [37], the development of a mesenchymal phenotype and the loss of E-cadherin expression are often associated with the expression of mesenchymal cadherins such as N-cadherin and/or Cad11. EMT is associated with the acquisition of migratory properties that promote metastasis. Interestingly, metastatic 786-O RCC cells in bone express a higher level of Cad11 than those in liver or lymph nodes, suggesting that Cad11 expression in Bo-786-O cells may support other functions uniquely required for bone metastasis in addition to migration. Consistent with such a possibility, previous studies on prostate cancer and breast cancer demonstrated that Cad11 contributes to bone metastasis [12], [13], [14], [15]. It is of interest to examine whether silencing Cad11 in Bo-786-O cells can decrease RCC bone metastasis. Our attempts to address this question were inconclusive as a majority of animals injected with Bo-786-O cells with or without knockdown of Cad11 did not survive long enough for further analysis of tumors in bone. We have performed x-ray, microCT, and histology on mice injected with 786-O cells in order to determine whether an osteolytic or osteoblastic reaction occurs, and did not detect obvious osteolytic lesions due to insufficient tumor growth in bone. This problem may be unique to 786-O cells, as we did not encounter such a problem when injecting mice with PC3-mm2 prostate cancer cells. Thus, whether an increase in Cad11 expression alone is sufficient to increase RCC bone metastasis requires further study.

CXCR4 is another adhesion molecule that has been implicated in the acquisition of invasive [38] and metastatic phenotypes in several cancer types, such as breast cancer [17], melanoma [39], prostate cancer [16] and renal cancer [40]. Studies have shown that higher CXCR4 expression is strongly associated with advanced RCC [41] and in RCC metastasis [42]. Our observations that CXCR4 expression was elevated in metastatic cell lines from bone and other organs, suggesting that CXCR4 may play a role in 786-O cells metastasis, but not specifically to the bone.

The hypervascularity of RCC is attributed to the mutation of the VHL tumor suppressor gene [21], [43]. Indeed, 786-O harbors an inactivating mutation in one VHL allele, while the second allele is deleted [44]. Our study revealed that the gene expression levels of angiogenic molecules such as HIF-1α and VEGF in 786-O cell lines were relatively high (data not shown). However, we did not detect significant differences in the gene expression among metastatic cell lines derived from organs. These results indicate that although angiogenesis plays an important role in the development and metastasis of RCC due to the loss of VHL function, it is not specific to bone metastasis. The angiopoietin-Tie-2 signaling axis is an alternative pathway to promote angiogenesis. However, the role of Ang-1 in tumor angiogenesis remains controversial. Some studies suggested that Ang-1 is angiogenic, whereas, others indicated that it inhibits angiogenesis, tumor growth and vascular permeability [6]. We found that *Ang1* message is decreased in organ-derived 786-O RCC cells (Fig. 4C). However, whether this leads to a decrease in protein expression was not examined. The significance of Ang1 in 786-O bone metastasis is not clear and therefore requires further study.

Bone lesions in patients with RCC are exclusively osteolytic [2], [3]. In many cancers, like breast and prostate cancers, tumor-produced growth factors or cytokines like PTHrP, RANKL, and IL-6 play important roles in bone osteolysis [29], [30], [31]. Contrasting evidence has been found. In the study of Weber et al. [45], although PTHrP is produced by bone-derived RCC cells, it did not appear to play a critical role in the cycle of bone destruction. Whereas, in the study of Strube et al. [46], PTHrP was highly expressed in metastatic cell lines suggesting that PTHrP might play a role in tumor-induced osteolysis similar to breast cancer bone metastasis. Additionally, it has also shown that RANKL did not substantially contribute to RANK-induced bone resorption [46]. In the current study, we found that gene expression of *PTHrP* and *IL-6* was significantly lower in bone-derived RCC 786-O cells than that in parental 786-O cells, and that *RANKL* gene expression in the 786-O RCC cells was too low to be detected. Our results agree with previous reports indicating that no *RANKL* mRNA expression was detected in human clear cell RCC cell lines, such as ACHN and Caki-1 cells [47], [48]. From these observations, we conclude that these tumor-produced factors may not play a critical role in affecting the metastasis of 786-O cells to bone. However, the possibility that these factors may be secreted as a result of interactions between 786-O RCC cells and bone marrow mesenchymal cells, and therefore may play a role in supporting the growth of RCC 786-O cells in bone, cannot be excluded.

Strube et al. [46] has also reported the selection of bone-derived metastatic 786-O cell lines through multiple cycles of in vivo selection. The highly selected cells showed strong osteolytic property with high levels of PTHrP [46]. As tumor cells are heterogeneous with ability to metastasize to various organ sites [8], we chose to use first generation of metastatic tumor 786-O RCC cell lines to determine the very initial factors that may involve in homing, retention and proliferation at bone site. Whether repeated in vivo selection enriched for the cells that express high levels of PTHrP is not clear.

In conclusion, among the several candidate factors examined, including angiogenic and osteolytic factors, we found that only one membrane protein, Cad11, was involved in organ-specific metastasis in bone using the 786-O cell line. Additional membrane proteins that are important for organ-specific targeting of metastatic RCC cells may be identified by using other RCC cell lines, and by other methods such as proteomics.

## Supporting Information

Table S1 Oligonucleotides of primers (Homo sapiens). The detailed information of primers used for real time PCR analysis was listed in Table S1.
(DOCX)
