# Supplementary material for: Cadherin-11 in Renal Cell Carcinoma Bone Metastasis
Source: PLoS One. 2014 Feb 24;9(2):e89880. doi: 10.1371/journal.pone.0089880 (PMC3933681; doi:10.1371/journal.pone.0089880)
Supplement: Table S1 — Oligonucleotides of primers (Homo sapiens). The detailed information of primers used for real time PCR analysis was listed in Table S1. (DOCX) [file pone.0089880.s001.docx]

Table S1. Oligonucleotides of primers (Homo sapiens)

| **Name** | **Forward (5'-3')** | **Reverse (5'-3')** | **Accession** | **Position** | **Size* (bp)** |
| --- | --- | --- | --- | --- | --- |
| Cadherin-11 | ACCCTCACCATCAAAGTCTG | TCAGGGTCACAAACAATACT | BC013609.1 | 1933-2095 | 160 |
| CXCR4 | AGCATGACGGACAAGTACAGG | GATGAAGTCGGGAATAGTCAGC | AF025375.1 | 211-519 | 309 |
| HIF-1α | TGCAACATGGAAGGTATTGC | CCAAGCAGGTCATAGGTGGT | AB733094.1 | 439-543 | 104 |
| VEGF-α | CTTGCCTTGCTGCTCTACCT | GCAGTAGCTGCGCTGATAGA | NM_003376.5 | 1072-1194 | 123 |
| Ang1 | GCAACTGGAGCTGATGGACACA | CATCTGCACAGTCTCTAAATGGT | [U83508](http://jcem.endojournals.org/external-ref?link_type=GEN&access_num=U83508) | 1059-1174 | 115 |
| Tie-2 | GATTTTGGATTGTCCCGAGGTCAAG | CACCAATATCTGGGCAAATGATGG | NM_000459.3 | 3386-3712 | 326 |
| c-MET | ATTTTGCTTTGCCAGTGGTGG | GAGCGATGTTGACATGCCACT | NM_001127500.1 | 2461-2622 | 161 |
| PTHrP | CGCCTCAAAAGAGCTGTGTC | TCTTTGTGTTGGGAGAGGGC | NM_002820.2 | 419-571 | 172 |
| IL-6 | GAAAGCAGCAAAGAGGCACTG | GCTCTGGCTTGTTCCTCACTA | NM_000600.3 | 351-541 | 191 |
| β-actin | GGGACCTGACTGACTACCTCA | GGGACCTGACTGACTACCTCA | BT019932 | 548-648 | 110 |

*: Production length of PCR

|  |  |  |  |  |  |
| --- | --- | --- | --- | --- | --- |
|  |  |  |  |  |  |
|  |  |  |  |  |  |
|  |  |  |  |  |  |
|  |  |  |  |  |  |
|  |  |  |  |  |  |
|  |  |  |  |  |  |
